# ExaBayes: Massively Parallel Bayesian Tree Inference for the Whole-Genome Era

**DOI:** 10.1093/molbev/msu236

**Published:** 2014-08-18

**Authors:** Andre J. Aberer, Kassian Kobert, Alexandros Stamatakis

**Affiliations:** ^1^Scientific Computing Group, Heidelberg Institute for Theoretical Studies, Heidelberg, Germany; ^2^Institute for Theoretical Informatics, Karlsruhe Institute of Technology, Karlsruhe, Germany

**Keywords:** software, Bayesian statistics, phylogenetic inference, whole-genome analyses, parallelization

## Abstract

Modern sequencing technology now allows biologists to collect the entirety of molecular evidence for reconstructing evolutionary trees. We introduce a novel, user-friendly software package engineered for conducting state-of-the-art Bayesian tree inferences on data sets of arbitrary size. Our software introduces a nonblocking parallelization of Metropolis-coupled chains, modifications for efficient analyses of data sets comprising thousands of partitions and memory saving techniques. We report on first experiences with Bayesian inferences at the whole-genome level using the SuperMUC supercomputer and simulated data.

The task of resolving the tree of life of extant species remains one of the grand challenges in evolutionary biology. As the number of trees grows superexponentially with the number of species for which an evolutionary tree is reconstructed, tree inference is considered a hard problem in computer science. The plethora of algorithmic challenges associated with phylogenetic trees and their efficient computation gave rise to the discipline of “phyloinformatics.” Likelihood-based statistical methods are highly popular because they can incorporate complex evolutionary models. Popular likelihood-based tools comprise methods for maximum-likelihood (ML) estimation, such as RAxML (Stamatakis 2014) and PhyML (Guindon et al. 2010), as well as Bayesian inference packages, such as MrBayes (Ronquist et al. 2012) and BEAST (Drummond et al. 2012). Likelihood-based methods can, in fact, unravel the true evolutionary tree given enough data and the appropriate model (Yang 1994b). Although the ML approach strives to optimize the likelihood of the tree and model given the data, Bayesian inference uses Markov chain Monte Carlo (MCMC) (Hastings 1970) sampling to integrate over the entire parameter space (e.g., model parameters and tree topologies) given the data and subjective prior assumptions.

Inexpensive wet-lab sequencing technologies allow amassing molecular evidence from hundreds of genes (DellAmpio et al. 2014). This amount of data is often required for resolving ancient radiations (Dunn et al. 2008). Hence, so-called phylogenomic data sets are being increasingly used to disentangle evolution. Collaborative efforts such as the 1K Insect Transcriptome Evolution (http://1kite.org, last accessed August 13, 2014) project aim at assembling the entirety of molecular sequence data to infer accurate phylogenies. Conducting such phylogenomic analyses is challenging due to exorbitant runtime and memory requirements. In a recent study (Rinke et al. 2013) with more than 2,000 microbial species and more than 8,000 amino acid alignment characters, the authors did not complement ML trees by Bayesian tree inferences. The reason for this was the limited ability of current Bayesian inference software to leverage supercomputer resources (often several CPU-months or CPU-years are required) and to accommodate the excessive main memory requirements which for this example are in the order of 10–20 GB.

## Novel Approaches and Software Features

We resolve these computational limitations of Bayesian inference tools by introducing ExaBayes, a software package engineered for efficient Bayesian tree inference on data sets of almost arbitrary size. ExaBayes can conduct Bayesian analyses for the most widely used priors, models, and input data types. These comprise Dirichlet, exponential and uniform priors, the general time-reversible (GTR) model of nucleotide substitution (Tavaré 1986), the Γ model of rate heterogeneity (Yang 1994a), and unconstrained branch length sampling. For protein data, we offer 18 commonly used fixed-rate substitution matrices as well as a GTR model. We also implemented a comprehensive set of topological proposals that assure rapid convergence and adapted these for massively parallel execution.

ExaBayes is a self-contained software package, that is, it comprises several postprocessing methods such as stand-alone tools for building consensus trees, for assessing topological convergence among independent runs (Lakner et al. 2008), and for extracting sample statistics. The output format is compatible with popular visualization tools, such as FigTree (Rambaut 2014) or Tracer (Rambaut and Drummond 2007).

Runtime as well as memory requirements of Bayesian inference is largely dominated by evaluating the phylogenetic-likelihood function. In ExaBayes, we deploy the efficient-likelihood kernel developed for RAxML (Stamatakis 2014) that allows fully leveraging the computational power of SSE and AVX vector units on modern CPUs. The sequential AVX version of ExaBayes outperforms MrBayes for DNA data, whereas for protein data MrBayes is faster (see Supplementary Material online, for detailed discussions on this and further results). The central contribution of ExaBayes lies in its scalability.

**Fig. 1.**
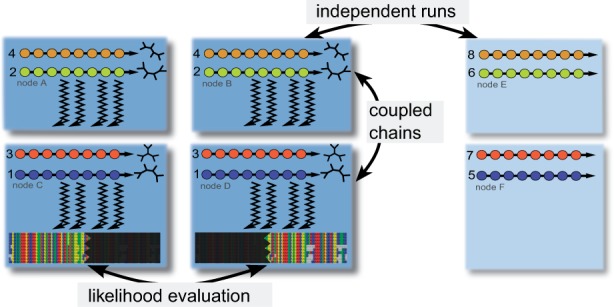
The three layers of parallelism employed by ExaBayes (distributed likelihood evaluation, distributed Metropolis-coupled chains, and distributed independent analyses).

**Fig. 2.**
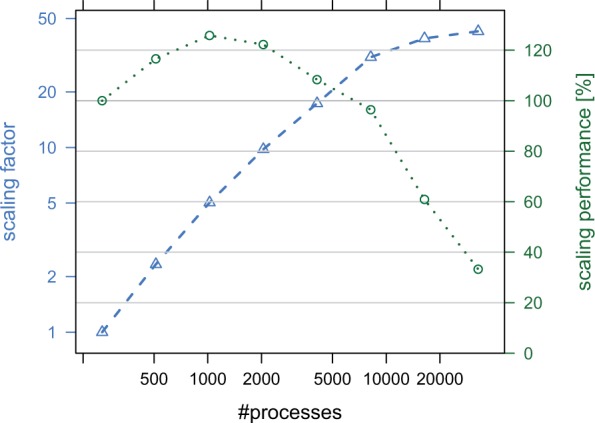
Scaling factor (sequential runtime divided by parallel runtime) and efficiency (scaling factor divided by number of processes) for executing ExaBayes on 256 cores up to 32,768 cores on a 200 species alignment with 500,000 characters.

ExaBayes implements three layers of parallelism (see fig. 1). For instance, the parallel version of ExaBayes allows to distribute the likelihood calculations across several processors and across several computing nodes in a cluster. Thus, the resources of an entire cluster or supercomputer can be used to accommodate the runtime and memory requirements of large-scale alignments. Parallel execution reduces runtimes almost linearly to the number of CPUs used. For a single chain and on a simulated data set with 200 species and 500,000 DNA characters, using the parallel version of ExaBayes reduces runtimes from 1 day and 4 h on one core to only 43.5 s on 8,192 cores, achieving a speedup of 2,368-fold. If we increase the number of characters by a factor of 10, ExaBayes scales from 256 up until at least 32,768 cores and improves runtime even faster than theoretically expected for up to 4,096 cores because of increased cache efficiency (see fig. 2).

ExaBayes implements Metropolis-coupling (Geyer 1992), a fundamental mechanism to accelerate convergence on “difficult” data sets. Heated chains with increased acceptance probabilities are coupled to the chain that is being sampled. Swaps between chains are accepted proportional to the posterior probability of chain states. As memory requirements increase linearly with each additional chain, large data sets require chains to be distributed across several CPUs or computing nodes in a cluster (second layer of parallelism). To this end, we modified the state-of-the-art parallel algorithm for Metropolis-coupled MCMC (Altekar et al. 2004) to use faster, nonblocking communication among processes (see supplementary material, Supplementary Material online, for a detailed description). Thus, when a set of processors propose a swap through a nonblocking message, they can immediately continue doing useful work by conducting calculations on a different chain that will not be swapped. This reduces runtimes by up to 19% and thus avoids wasting hundreds of CPU hours for large-scale analyses. Finally, independent analyses can be executed concurrently using a third layer of parallelism (see fig. 1).

Moreover, ExaBayes adapts two orthogonal memory saving techniques. The so-called subtree equality vector approach allows to save memory proportional to the fraction of unknown or missing data. At the same time, it can also accelerate likelihood calculations on data sets with a large proportion of missing data. This strategy was designed for phylogenomic data sets (Izquierdo-Carrasco et al. 2011), where unknown orthologs can lead to a large fraction of missing data exceeding for instance 75% (DellAmpio et al. 2014). Our second technique allows discarding memory-intensive partial results by recomputing these on demand. Three settings trade varying amounts of runtime for additional memory savings by means of recomputation. Specifically, when coupled chains are distributed, this reduces memory requirements by a factor of up to 2, while we observe a slow-down of less than 1.5-fold. As the size of data sets that can be computed by ExaBayes is merely constrained by available hardware, our memory saving techniques allow conducting ambitious analyses on small and less expensive computer clusters.

For improved model fit, it is often desirable to sample distinct model parameters (e.g., substitution rates) for each gene or partition of an alignment. We have tested ExaBayes using simulated alignments with 1,000–10,000 partitions. All parameters (including branch lengths, but excluding the topology) can be flexibly linked or unlinked across partitions. We observed a performance decrease with an increasing number of partitions. To alleviate this issue, we modified our MCMC algorithm and data-to-processor assignment scheme. This modification induces a runtime improvement of up to 22 times for runs in which branch lengths are linked across all partitions. ExaBayes runs up to 87 times faster, when each partition has distinct branch lengths (i.e., branch lengths are unlinked across partitions).

## Inference from a Whole-Genome Data set

To demonstrate the capabilities of ExaBayes, we executed a Bayesian inference on a “difficult” complete simulated genome (for which the “true” evolutionary history is known) comprising 100 partitions with 1,000,000 characters each (inspired by a per-chromosome partitioning). To simulate the alignment, we used a tree with 200 species. The tree is bush-like and contains many long, outer branches and short, hard-to-resolve, inner branches. As the RAM requirements of this data set exceed 5 TB, we employed the SuperMUC supercomputer, which is currently among the ten fastest supercomputers in the world. A total of four independent runs with one chain each seeded by reasonable (i.e., nonrandom) parsimony starting trees (Fitch and Margoliash 1967) rapidly converged to the true tree topology within less than 20,000 generations (total chain length: 100,000 generations; a sample was extracted every 500 generations). All branches showed 100% certainty (posterior probability). Using over 4,096 CPU cores, the slowest run took 1 h 40 min. Thus, the accumulated CPU-time over all four runs is 3 years and 45 days. To avoid potential biases induced by the parsimony starting trees, we also ran two independent chains starting from random trees. Here, the chains converged to the true tree topology after ≈60,000 generations. With random starting trees, we have to discard substantially longer burn-ins, before we attain an accurate sampling of the posterior probability of the trees.

**Fig. 3.**
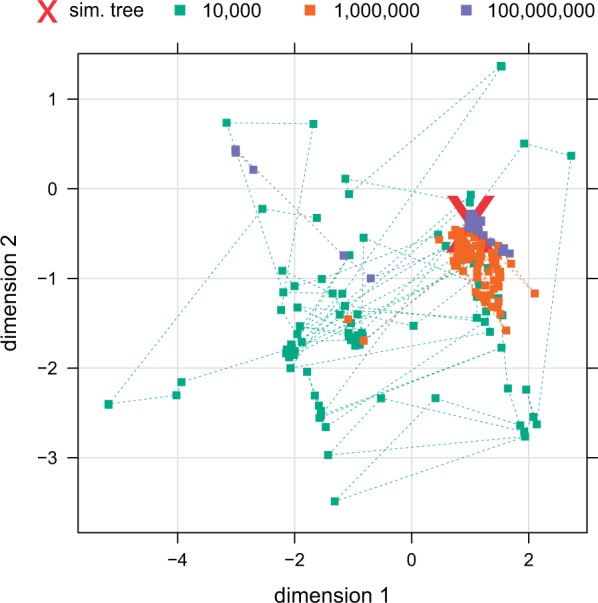
Inference from genome-sized data: 2D-rescaled representation employing multidimensional scaling (MDS) of Robinson–Foulds distances among sampled trees for chains with varying amount of data. The position of the simulated true tree is shown in red. The applied MDS algorithm maps identical trees to adjacent nonidentical positions (i.e., overlapping squares represent identical trees).

We examined how the posterior probability of the trees changes as we reduce the amount of data in steps of 2 orders of magnitude. If we discard 33% of samples as burn-in, we obtain 1) 100% confidence for all splits in the tree inferred from 100,000,000 characters, 2) only one split with 98.5% certainty for 1,000,000 characters, and 3) nine splits with a posterior probability between 61.2% and 99.2% for 10,000 characters. For 10,000 characters we do not attain maximum confidence in the true tree, even if the burn-in phase is substantially extended. Thus, we conclude that this amount of data is insufficient for inferring a reliable tree on this data set. As illustrated in figure 3, the chain instead jumps between several trees of high posterior probability after burn-in.

Finally, we examined how confidence intervals for branch lengths change as we increase the amount of data. We find that, with only 10,000 characters, we mostly do not obtain accurate branch length estimates and that confidence intervals cover a wide spectrum specifically for short branch lengths. Branch lengths can be determined more accurately with more data; however, increasing the data set size from 1,000,000 characters to 100,000,000 characters does not substantially decrease the standard deviation in branch length samples (see supplementary material, Supplementary Material online). We observed that genome-scale data allow for high certainty about the “true” (here simulated) branch length; however, even this amount of data does not guarantee small confidence intervals for branch lengths in the range of $10^{-3}-10^{-4}$. Nevertheless, we expect that with chromosome- and genome-size data, extremely precise estimations of divergence times can be performed.

## Supplementary Material

Supplementary material is available at *Molecular Biology and Evolution* online (http://www.mbe.oxfordjournals.org/).

Supplementary Data
