# Supplementary material for: ExaBayes: Massively Parallel Bayesian Tree Inference for the Whole-Genome Era
Source: Mol Biol Evol. 2014 Aug 18;31(10):2553–6. doi: 10.1093/molbev/msu236 (PMC4166930; doi:10.1093/molbev/msu236)
Supplement: Supplementary Data [file supp_31_10_2553__index.html]

ExaBayes: Massively Parallel Bayesian Tree Inference for the Whole-Genome Era — ExaBayes: Massively Parallel Bayesian Tree Inference for the Whole-Genome Era — ExaBayes: Massively Parallel Bayesian Tree Inference for the Whole-Genome Era — Supplementary Data 

# ExaBayes: Massively Parallel Bayesian Tree Inference for the Whole-Genome Era

## Supplementary Data

files

**Files in this Data Supplement:**

- Supplementary Data - zip file
