# Supplementary material for: ExaBayes: Massively Parallel Bayesian Tree Inference for the Whole-Genome Era
Source: Mol Biol Evol. 2014 Aug 18;31(10):2553–6. doi: 10.1093/molbev/msu236 (PMC4166930; doi:10.1093/molbev/msu236)
Supplement: Supplementary Data [file supp_msu236_suppl_data.zip › mbe-supplement.pdf]

Supplement to "ExaBayes: Massively Parallel Bayesian Tree Inference for the Whole-Genome Era"

Andre J. Aberer*,[1], Kassian Kobert[1] and Alexandros Stamatakis[1,2]
[1]Scientific Computing Group, Heidelberg Institute for Theoretical Studies, Germany
[2]Institute for Theoretical Informatics, Karlsruhe Institute of Technology, Karlsruhe, Germany
***Corresponding author:** E-mail: andre.aberer@h-its.org
Associate Editor:

Abstract

In this supplement, we provide an extended description of the implementation of `ExaBayes`, describe and evaluate the parallelization scheme, compare its sequential performance to `MrBayes` and verify the simulation outcome against `MrBayes`. We explain and evaluate techniques for reducing the memory footprint of large-scale analyses and techniques that improve the efficiency of highly-partitioned analyses. Finally, we provide supplementary information about the simulated whole-genome inference.

Key words: Software, Bayesian statistics, Phylogenetic Inference, Whole-Genome analyses, Parallelization.

Extended Software Description

`ExaBayes` (version 1.2.1) supports x86 systems running Linux or MacOS. For the sequential version, we distribute binary executables without external dependencies. `ExaBayes` is available under `http://exelixis-lab.org/web/software/exabayes/`. The parallel version requires a working *Message Passing Interface* (MPI) installation (usually simple to install via package management and always available on computer clusters). The source code includes distributions of all external software dependencies, that is, the Nexus class library (Lewis, 2003) and the random number library Random123 (Salmon *et al.*, 2011).

`ExaBayes` is fully checkpointed. Thus, an analysis can be interrupted (e.g., because of walltime restrictions on large clusters) and later be resumed from the previous checkpoint. In addition, `ExaBayes` comes with a strong guarantee of reproducibility, that also extends to checkpointing. Our implementation pursues the goal that for an identical configuration and identical random number seed, `ExaBayes` produces identical chains regardless of (i) the likelihood implementation being used, (ii) the data distribution scheme, (iii) the parallel configuration or (iv) an arbitrary number of restarts from checkpointing files. While the MPI standard does not guarantee that rounding errors will not affect distributed summation operations, we obtained identical chains for up to 32,768 processes.

A substantial problem for highly parallel runs are runtime phases that cannot be executed

Suppl.

For permissions, please email: journals.permissions@oup.com

in parallel. Thus, CPU-time is possibly lost thousand-fold in non-parallelized code regions and might diminish the available CPU time budget without properly using it. In ExaBayes, we reduced this sequential overhead to a minimum: we provide a pre-processing utility for parsing the alignment file (which can easily exceed 1 GB for resource-intensive runs). The resulting pre-processed binary file substantially reduces start-up time (for instance by 89% for a file of 950 MB). Furthermore, using our stand-alone topological convergence tool makes it easy for the user to execute independent analyses in small batches in parallel and refer the test for topological convergence to a single process.

Evolutionary Models

ExaBayes allows for tree inference from partitioned alignments, in other words, a sequence dataset can be divided into several partitions and model parameters are estimated separately for each data partition. ExaBayes focuses on one of the standard models of substitution in molecular phylogenetics, the *general time-reversible* (GTR) model (Tavaré, 1986). ExaBayes allows to integrate over free parameters (state frequencies and substitution rates) of DNA or amino acid (AA) GTR matrices. Alternatively, AA partitions can be configured, such that ExaBayes integrates over 18 fixed-rate empirical matrices (e.g., WAG (Whelan and Goldman, 2001) or LG (Le and Gascuel, 2008)), for which state frequencies are either specified by the model or ExaBayes integrates over the 19 free parameters.

The discretized Γ model of rate heterogeneity (Yang, 1994) accounts for varying evolutionary rates across sites. Here, ExaBayes integrates over the α shape parameter of the Γ distribution.

All of these parameters (as well as the branch lengths) can be linked arbitrarily across partitions (i.e., a partition may share the same α parameter with one set of partitions, while sharing state frequencies with a different set of partitions).

Proposals

For the integration over the parameter space, we implemented an analogous set of proposals as available in MrBayes. For continuous parameters, we deploy sliding window proposals, proposals drawn from a scaled Dirichlet distribution, and multiplier proposals. All proposals for continuous parameters are tuned. In other words, the proposal mechanism adjusts its parameters, such that proposals are neither too bold nor too modest and after a sufficient number of proposals a desirable integration efficiency is attained.

ExaBayes implements metropolized versions of the topological proposals discussed in an evaluation of topological proposals of MrBayes (Lakner *et al.*, 2008), namely the stochastic nearest neighbor interchange (stNNI), extending subtree pruning and regrafting (eSPR), extending tree bisection (eTBR) and parsimony-guided subtree pruning and regrafting (pSPR) proposals. We modified eSPR and eTBR, such that their

proposal density ratio is 1. In the default version of the eSPR, the pruned subtree traverses descendants of the remaining subtree until a stochastic criterion is met or an external branch is encountered. This tip-specific behavior needs to be accounted for in the proposal density ratio. In our move, the subtree does not necessarily stop at the external branch, but may continue traversing into the opposite direction. Thus, no modification to the proposal density ratio is necessary. This can easily be shown by demonstrating that for each forward and backward move the number of paths between the pruning and the insertion branch is identical and must be equally probable.

The original pSPR prunes a subtree and proposes regrafting at a branch that is drawn according to a score derived from the parsimony score (Fitch *et al.*, 1967) at the insertion position. Computation of parsimony scores are orders of magnitude faster than likelihood computations. However, we have to determine the parsimony score of a large number of possible regrafting positions (e.g., almost 2,000 positions for a tree with 1,000 species), although reinsertion most likely takes place in the topological neighborhood of the pruning branch. To account for that, we implemented a modified version that only considers branches for regrafting that are within a radius of n (either specified by the user or $n := \lfloor 2 \cdot \log(t) \rfloor$, where t is the number of taxa in the tree) around the pruning branch (which depending on the dataset may increase the length of the burn-in phase by a factor of two, but often reduces runtime by 10%). We also parallelized the parsimony evaluation. Thus, in the parallel version of `ExaBayes`, two additional communication steps are necessary for the pSPR: one for the parsimony evaluation within the radius of the pruning location for determining the reattachment location, and one for determining the probability of the backward move.

Adaptations to proposals on GTR matrices

For the internal likelihood computation, `ExaBayes` represents GTR rates relative to one rate (which assumes a value of 1.0). In case of DNA data, the reference rate is $r(G \rightarrow T)$. Furthermore, internally branch lengths are represented relative to the mean substitution rate (that is dependent only on the state frequencies and substitution rate in the aforementioned representation). As a consequence, updating state frequencies and substitution rates also modifies the overall branch lengths (which needs to be considered in the proposal density ratio and prior ratio of the affected proposals). Thus, proposals integrating over state frequencies or substitution rates effectively also integrate over the tree length. Using the proposal mixture as applied in `MrBayes`, we noticed that `ExaBayes` sampled tree lengths much more efficiently than `MrBayes`, however substitution rates were not sampled sufficiently. Thus, in `ExaBayes`, we reduced the weight for our tree length multiplier proposal by 50% and instead doubled the weight for the sliding window and

the Dirichlet proposal on substitution rates. While this allows for good overall sampling efficiency, the reference rate is still sampled less efficiently in the worst case (see **Sect.** *verification*). This is because updates on the reference rate heavily affect the mean substitution rate and in turn leads to higher absolute values of the proposal density ratio.

Furthermore, we found that even with tuning, proposals usually applied to DNA GTR matrices perform sub-optimally on AA GTR matrices. An AA GTR matrix has 189 free parameters. Thus, a tuned Dirichlet proposal that modifies all values in one step, will only apply modest updates that do not change values substantially. A sliding window proposal that updates one or two rates can perform a bolder update, this however only affects a negligible proportion of the matrix. As a compromise between both alternatives, we implemented a Dirichlet proposal that modifies all substitution rates associated with one specific amino acid (i.e., the proposal updates 19 values at a time). The proposal density ratio of this proposal directly follows from the Dirichlet densities of the affected rates.

For our three amino acid datasets, we ran chains for 1,000,000 generations using three different proposal mixtures to evaluate this *rate-specific Dirichlet* proposal (5 chains per strategy). In our scenario, the relative number of updates on the substation rate parameter amounts to 4.44%. The default proposal mixture (strategy 1) applies 50% sliding window and 50% Dirichlet proposals. For strategy 2, we added the rate-specific Dirichlet proposal and applied all three proposals with equal probability. In strategy 3, only the rate-specific Dirichlet proposal is applied.

As shown in **Fig.** 1 exclusive usage of the rate-specific Dirichlet proposal leads to increased sampling efficiency for 2 out of 3 datasets, however it performs worse than the standard strategy on the 72-taxon dataset. Strategy 2, which adds the rate-specific Dirichlet proposal to the default proposals leads to increased sampling efficiency on all three datasets.

Parallelization Scheme

Top-Level Description

Bayesian phylogenetic inference is suited for parallelization at three distinct program layers:

- *data-level:* calculation of log-likelihood / parsimony score of characters of an alignment,
- *chain-level:* computation of the generations for each chain in a set of Metropolis-coupled chains,
- *run-level:* inference using multiple independent analyses/runs (each sampling the parameter space using a cold chain, possibly coupled to further heated chains).

In `ExaBayes`, we implemented all three levels of parallelism using MPI (Message Passing Interface). This allows users to run Bayesian sampling using an arbitrary number of processes under almost any combination of the three layers of parallelism. For instance, given 128 processors, the user can choose to run 4 analyses in parallel

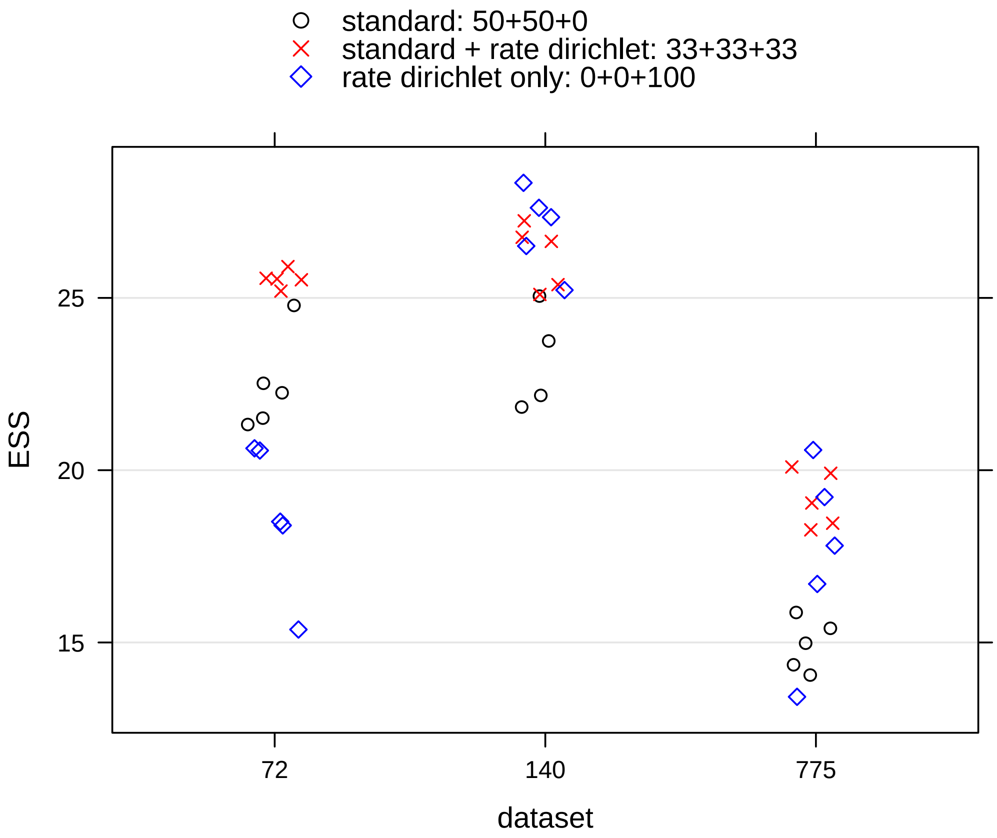

FIG. 1. Sampling Efficiency of AA GTR proposals: Effective sampling size (ESS) averaged over all amino acid substitution rates for three different proposal mixtures. For each strategy the proportion of proposals spent on (i) sliding window proposals, (ii) Dirichlet proposal and (iii) rate-specific Dirichlet proposals is given.

with 4 parallel Metropolis-coupled chains each, and assign 8 processes to each coupled chain for the likelihood calculation. Currently, the only limitation to flexibly combining layers of parallelism is that `ExaBayes` prohibits configurations inducing load imbalance that would severely reduce parallel efficiency (e.g., with a total of 3 coupled chains it is not possible to assign 2 chains to one group of processes and only 1 chain to another group).

Parallelism is modeled similar (yet not identical) to a 3-dimensional Cartesian grid. We assign three coordinates (x_1,x_2,x_3) of a 3D grid to each processor. Thus, all processes with identical x_1 are assigned to the same set of independent analyses. Processes with identical x_1 *and* x_2 in turn all contribute to the calculation of the same generation of the same coupled chains that belong to an independent analysis. x_3 specifies which portion of the data is evaluated by a process. If the number of processes is not a multiple of the product of all dimension lengths, then we choose the largest Cartesian grid possible and distribute remaining processes equally among different partitions of a dimension in a recursive manner from run-level over chain-level to the data-level.

Data-Level parallelism.

Description. For phylogenomic datasets, data-level parallelism carries the main burden of parallel execution. Many processes evaluate a small portion of the alignment. Good parallel efficiency can oftentimes be achieved for as few as 100 alignment patterns, thus for a concatenation of 20 genes (assume 1,000 base pairs per gene), we already can employ 100 cpu cores efficiently. For data parallelism `BEAST` employs an OpenMP parallelization using the Beagle library (Ayres *et al.*, 2012) using multi-core machines or graphic processors (GPU). Beagle-GPU is also used by `MrBayes`. For phylogenomic datasets, this approach limits the scalability of a parallel analyses in two ways: (i) we can not employ more processes than available on a *single* computing node (often 12 or 16 processes) or GPU and (ii) memory requirements of the alignment must not exceed the available main memory. All data-level parallelizations in `BEAST`/`MrBayes` correspond to a master-worker scheme: a single process governs the execution of the main algorithm and instructs worker threads to perform likelihood evaluations.

We previously demonstrated (Stamatakis and Aberer, 2013) in the context of maximum likelihood (ML) inference, that such a master-worker scheme does not scale well to several hundreds of cpu-cores. As an alternative, we introduced `ExaML`, where the tree search algorithm is executed by every process, which severely reduces expensive communication costs among processes for likelihood evaluations. In `ExaBayes` we use a modified version of `ExaML` for likelihood computations. We fully retained source code compatibility with `ExaML`, such that future advancements in `ExaML` easily can be integrated in `ExaBayes`.

Evaluation. For evaluation, we sub-sampled the simulated whole-genome alignment to obtain a dataset D^{5e5} with 500,000 characters and a dataset D^{5e6} with 5,000,000 characters. The memory requirements of a Bayesian analysis for these datasets are roughly 24 GB and 240 GB, respectively. We measured the strong scaling (i.e., increasing the number of processors while keeping the input size fixed) capabilities of `ExaBayes` and quantify it by the *parallel speedup* and *parallel efficiency* metrics. Parallel speedup is defined as the ratio of the execution of the fastest sequential implementation (fT_1) and the execution time with n processes (T_n). Parallel efficiency measures whether speedup increases proportionally to the number of processes employed (it is thus defined as the speedup divided by the number of processes). For determining both measures, we ran one chain for 100,000 generations without partitioning, using default settings on the SuperMUC supercomputer (9,216 computing nodes consisting of 4 Intel Sandy Bridge processors with 4 cores each, thus 16 processes per node in total; 32 GB

RAM per node; 24 GB typically available to users). As sequential reference, we used the AVX version of `ExaBayes`, serving as the currently fastest-known implementation on DNA data (see **Sect.** *sequential performance*). D^{5e5} is the largest alignment that still can be computed on a single SuperMUC computing node. For D^{5e6}, we instead report scaling factors/efficiency that indicate how well the code scales compared to reference cases that are already executed on several computing nodes. We measured parallel execution times for numbers of processes that are a power of two (from 16 to 16,384 for D^{5e5} and from 256 to 32,768 for D^{5e6}).

Fig. 2 shows parallel speedup and efficiency for a chain with 100,000 generations on D^{5e5} and number of processors that is a power of 2 (from 16 to 16,384). We obtain a maximum parallel speedup of 2,368 with 8,192 processes. In absolute terms this means that the runtime could be reduced from 103,191 sec ($\approx$ 1 day, 4 hours) in the sequential case to 43.57 sec with more than 8,000 processors. The runtime cannot be decreased beyond this point, since with 16,384 processors, each processor works on less than 31 characters and thus parallel overhead becomes runtime dominating. Parallel efficiency initially decreases (since in the sequential case no communication is required at all), then increases (because of improved cache usage) and peaks at 128 processors, before parallel overhead reduces parallel efficiency again. Thus, with 128 processors the runtime can be reduced to $\approx$1,010 sec, while the loss of computational resources due to overhead is minimal.

Memory requirements of large alignments often exceed the available main memory on a computing node. In these instances, it is not possible to determine the sequential runtime fT_1. Instead, we use a $t_{n'}$ (with $n' < n$) as reference and report *scaling factors* and *scaling efficiency* accordingly. For **Fig. 2** (main text), we simulated an 200-taxa alignment with 5,000,000 bp (10 times larger than the aforementioned dataset). The increased size of the alignment allows the code to scale up to more than 32,000 processes achieving a scaling factor of 42.5. As for the smaller dataset, the scaling efficiency peaks as the number of processes increases, here at 1,024 processes.

Chain-Level Parallelism

Description. `ExaBayes` implements Metropolis-coupled MCMC (Geyer, 1992), also referred to as MC^3. MC^3 modifies the acceptance probability of chains by adding an exponent β to prior ratio and likelihood ratio and thereby increases the probability for heated chains to accept proposed states with low posterior probability. The temperature β for chain i (where $i := 0$ for the cold chain) is determined as,

$$\beta = \frac{1}{1 + i \cdot \delta},$$

where δ is a constant that can be provided by the user.

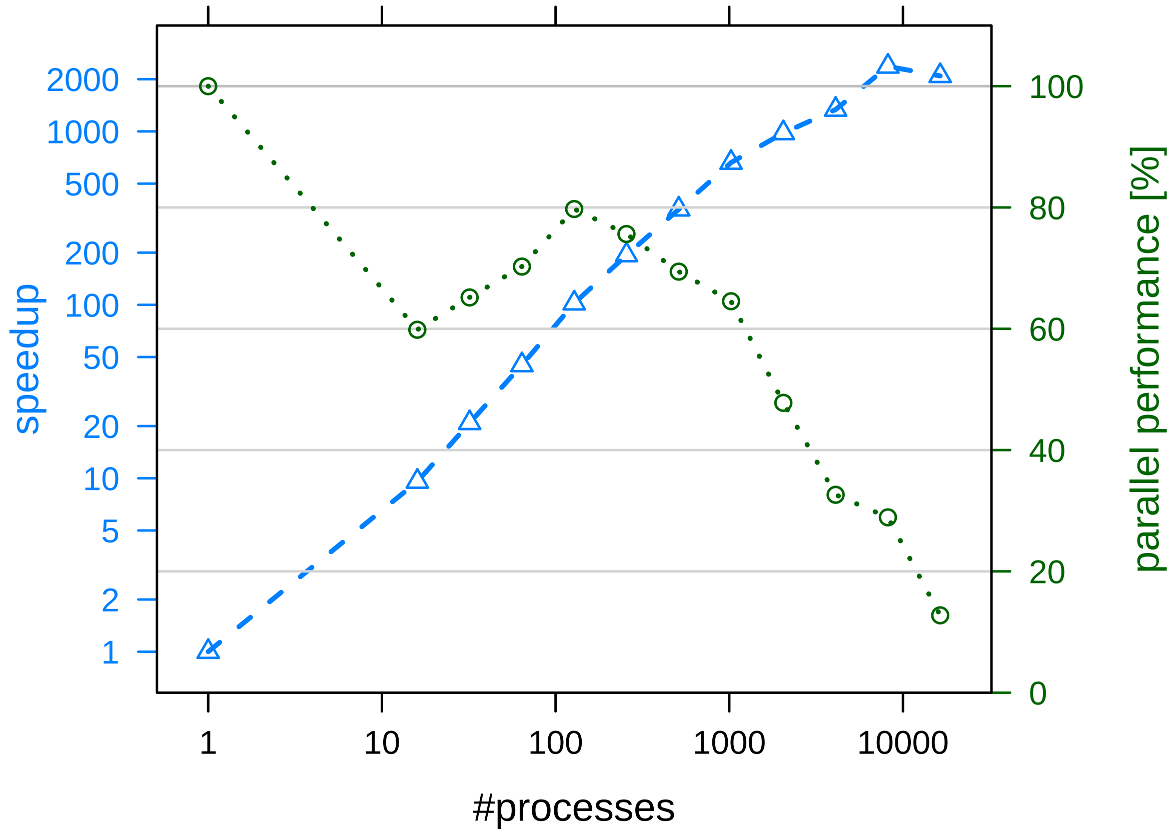

FIG. 2. Parallel Efficiency: Speedup (on log-scale) and parallel efficiency for 100,000 generations of a MCMC chain on a dataset with 200 taxa and 500,000 bp. Aside from the sequential case (1 process), the number of processes are powers of 2 (starting at 16, ending at 16,384) on the logarithmic x-scale.

For parallelization of Metropolis-Coupling, we extended the chain-level parallelization introduced in `MrBayes` (Altekar *et al.*, 2004) for the case where several processes compute the state of a single chain: each process instantiates an analysis-specific random number generator and can thus pre-compute all swap attempts at any generation number for the entire analysis. Processes compute generations of all chains assigned to them. If a process is not assigned to any chain of a swap attempt, this event can be ignored by the process. *Local* swaps (i.e., both involved chains are assigned to the process) can be executed without any interaction with other processes. *Remote* swaps (i.e., one local chain swaps with a remote chain that is computed by a different set of processes) are problematic for parallel efficiency. In this case, the set of processes working on one chain has to wait for the set of processes working on the other chain to complete. For a remote swap attempt in the original parallel algorithm, processes of both groups then exchange the posterior probability and their heat value. If the swap is accepted, the identity (e.g., *hottest chain*) of the remote chain is assumed. With the introduction of

tuned proposals, chains have to swap their entire sets of proposals including acceptance ratio counters, which drastically increased the amount of information to be communicated among processes. Our blocking implementation (processes are stalled until communication takes place) of this algorithm uses broadcast operations with MPI Intercomms to conduct the swap.

Instead of a fixed number of swap attempts, that takes place after a fixed number of generations, we draw the number of swap attempts from a binomial distribution: for a user-specified expected number of swaps per generation μ and a number of coupled chains c, we draw the number of attempted swaps x from a binomial distribution

$$x = \begin{cases} \mathrm{Bin}(c, \frac{\mu}{c}), & \text{if } \mu < 1 \\ \mathrm{Bin}(2c\mu, \frac{1}{2c}), & \text{else.} \end{cases}$$

Furthermore, we implemented a non-blocking version of the Metropolis-Coupling algorithm (see **Fig.** 3 for an example that compares the blocking to the non-blocking algorithm). Non-blocking communication allows processes to initiate a request for communication, to continue computation and regularly check the state of the request. Processes compute generations of a chain until a remote swap is required. Then, all processes assigned to the swapping chain use a non-blocking send operation to communicate the chain information to a peer process (or two peers if the number of processes assigned to both groups is not identical) working on the remote chain and initiates a request to receive the information from its peer. Thus, processes can continue to compute generations for other chains assigned to them. If a process does not have any outstanding generations it checks pending communication requests and synchronizes with all processes working on the same chains to ensure a deterministic execution.

Evaluation. A straight-forward way of demonstrating scalability of chain-level parallelism is to analyzing its weak-scaling properties (as performed by Altekar *et al.*). Here, the amount of work per process is kept fix (i.e., each process is assigned one of the n coupled chains) and we examine the ratio of sequential runtime (employing a single chain) to the runtime with p processes working on p chains (where one swap is attempted once all chains have progressed by 1 generation). As illustrated in **Fig.** 4 on a dataset with 150 taxa and 1,269 characters, the implementation in `ExaBayes` shows a minor speed advantage over `MrBayes`, mostly because of the aforementioned modification to draw the number of swaps per generation at random. While linear scaling could be demonstrated for `MrBayes` (Altekar *et al.*, 2004), in our experiment neither `MrBayes` nor `ExaBayes` exhibit linear scaling. We assume this is because of the severely higher communication effort that is necessary for chains with tuned proposals. In **Fig.** 4, we also included runtimes for the case that 2 processes are assigned to each chain. For 2 processes per

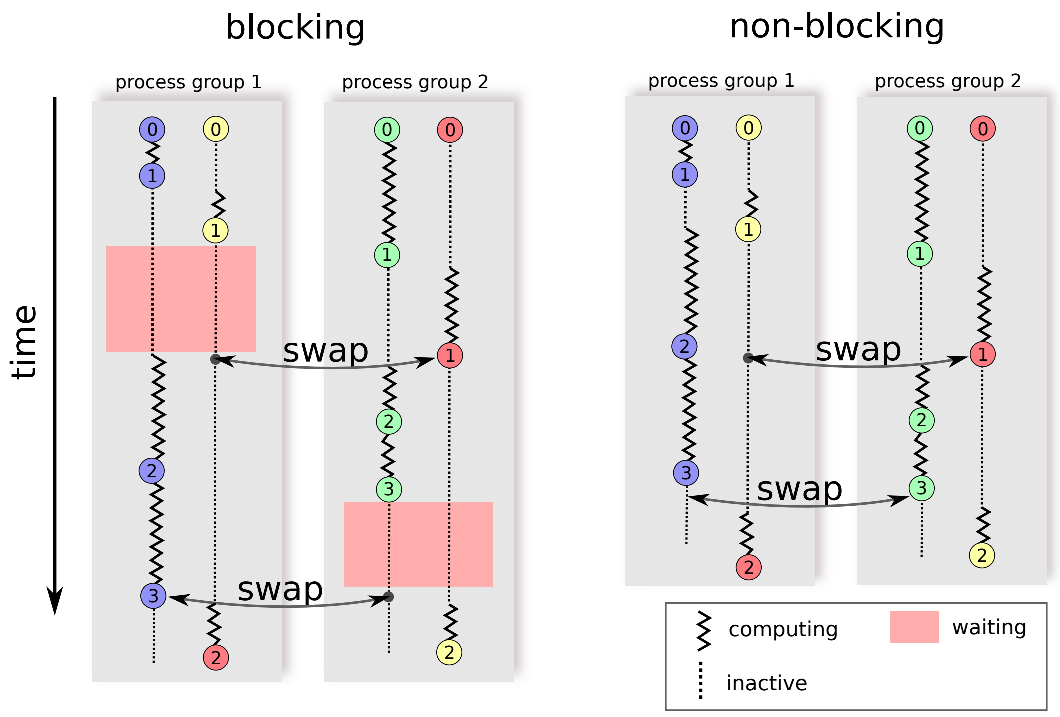

FIG. 3. Comparison between the blocking (*left*) and the non-blocking (*right*) algorithm for Metropolis-coupled MCMC. Two distinct groups of processes compute a total of 4 coupled chains (i.e., two chains are computed in parallel and 2 chains are assigned to each group). The color of a chain represents its heat increment (e.g., blue chain is the non-heated chain), numbers inside circles indicate the generation of the respective chain. In this scenario, the yellow and the red chain successfully swap after generation 1 and the green and the blue chain attempt a swap after generation 3.

chain, the runtime is close to 2× as fast (however, scaling efficiency is lost because of the increased need for synchronization among processes).

A major short-coming of this weak scaling plot is, that the number of swap attempts is limited to an average of one per generation. Simple addition of heated chains to a set of coupled chains with fixed heating scheme and number of swaps can be highly detrimental to the overall algorithm's performance (Atchadé *et al.*, 2010) (aside from increasing runtime). For instance, with 32 coupled chains, 1 swap attempt per generation and the default heating scheme (as also employed in `MrBayes`), a cold chain was only involved in 92 successful swap attempts in 50,000 generations. Thus, it becomes increasingly unlikely that any information from hotter chains is propagated to the cold chain.

Thus, in **Fig.** 5, we compare the scaling efficiency of 16 coupled chains running on 32 processes for increasing chain-level parallelism and for an increasing number of swap attempts per generation. As expected, scaling efficiency decreases as the number of swap attempts per generation are increased. We have chosen a small simulated dataset that comprises 200 taxa and 5,000 characters. Since this dataset is too small for 16 processes, we observe sub-optimal parallel efficiency at the left-hand side of **Fig.** 5, where only data-level parallelism is employed (i.e., all 16 processes evaluate part of the alignment and no remote swaps occur). As more and more coupled

chains are executed in parallel (to the point where each chain is executed in parallel by 2 processes at the right-hand side of **Fig.** 5), efficiency of data-level parallelism increases, since every process has a sufficient amount of work. With increased chain-parallelism however, processes spend more time waiting for remote chains to complete.

This effect is substantially alleviated for our non-blocking implementation. In the best case, the non-blocking version improves the parallel efficiency by 10.3%. In absolute terms this means that the non-blocking version reduces the runtime by 18.6%, a difference that easily can amount to several hundred cpu hours for phylogenomic datasets. The non-blocking implementation consistently outperforms the blocking version, even in extreme cases such as 16 swap attempts per generations (i.e., each chain is expected to swap 2 times per generation).

Sequential Performance

`ExaBayes` implements a set of proposals that is similar enough to the proposals implemented in `MrBayes`, such that a fair runtime comparison between the two tools is feasible. To achieve this, we have to set the radius parameter of the parsimony-guided topological SPR proposals to a number that covers the entire tree. The sequential runtime performance is vastly dominated by likelihood evaluations (typically about 95%). Among the remaining factors that affect the runtime, the parsimony score computation deserves mentioning. In `ExaBayes` parsimony computations account for a particularly low fraction of total runtime (typically 1-2%), since `ExaBayes` integrates the — to the best of our knowledge — fastest publicly and freely available parsimony implementation (Alachiotis and Stamatakis, 2011, see also: `https://github.com/stamatak/Parsimonator-1.0.2`).

Thus, given the comparability of the proposal mechanisms, the runtime assessment essentially boils down to a comparison of the underlying likelihood implementations. For `ExaBayes` as well as `MrBayes` a number of different versions are available. The native likelihood implementation in `MrBayes` is SSE3-vectorized and employs single-precision floating point arithmetics. While conducting numerical operations on single-precision floating point numbers is faster and requires 50% less memory than under double-precision, using single precision comes at the cost of reduced numerical accuracy of likelihood computations. Particularly in the context of maximum likelihood tree searches, the reduced numerical range can lead to problems with numerical stability (Berger and Stamatakis, 2010). Here, searches for trees with more than 1,000 taxa can become unfeasible. While an analysis of the numerical stability of Bayesian inference programs under single-precision arithmetics is outside the scope of this study, we emphasize that we did not encounter any issues pertaining to numerical stability

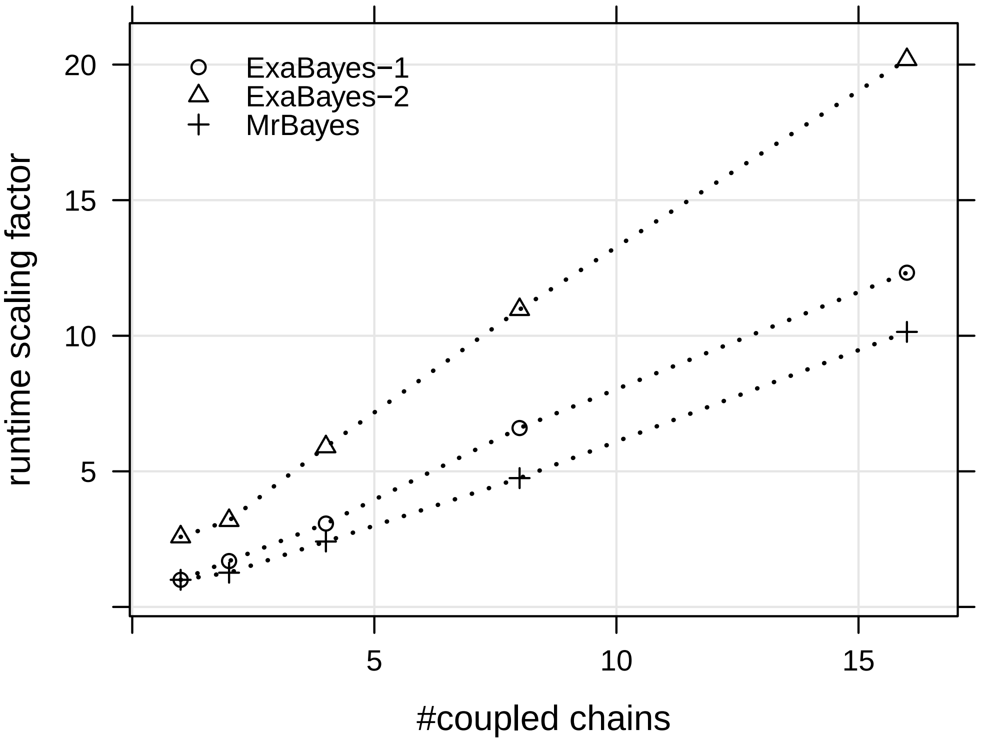

FIG. 4. Comparison of weak scaling at chain-level between ExaBayes and MrBayes. Number of coupled chains (all executed in parallel) on the x-axis. Y-axis shows ratio of sequential runtime of a single chain of either ExaBayes or MrBayes and the runtime with n coupled chains. We employed 1 process per chain in *ExaBayes-1* and 2 processes per chain in *ExaBayes-2*.

with MrBayes. Note that, alternatively, MrBayes can also be configured to employ either single- (non-vectorized) or double-precision (vectorized) floating point numbers when using the BEAGLE library for likelihood calculations, instead of its own, native implementation.

ExaBayes employs the highly optimized likelihood implementations (all double-precision) provided by RAxML (Stamatakis, 2014). Apart from a SSE-optimized version, ExaBayes also comes with an AVX-optimized version that makes full use of modern vector processing units as offered by recent x86 CPUs. Additionally, RAxML offers a likelihood implementation that omits the computation of conditional likelihoods for subtrees that entirely consist of undetermined or gap characters (while still yielding the correct likelihood) using subtree equality vectors (Izquierdo-Carrasco *et al.*, 2011) (SEV). This extension (which can be combined with either the SSE or AVX version) is particularly useful for large phylogenomic datasets consisting of several concatenated genes that usually exhibit a substantial proportion of missing data (e.g., due to the unavailability of orthologs). For instance, the proportion of undetermined/missing data in the largest super-matrix in a recent study (DellAmpio *et al.*, 2014) is 78.68%.

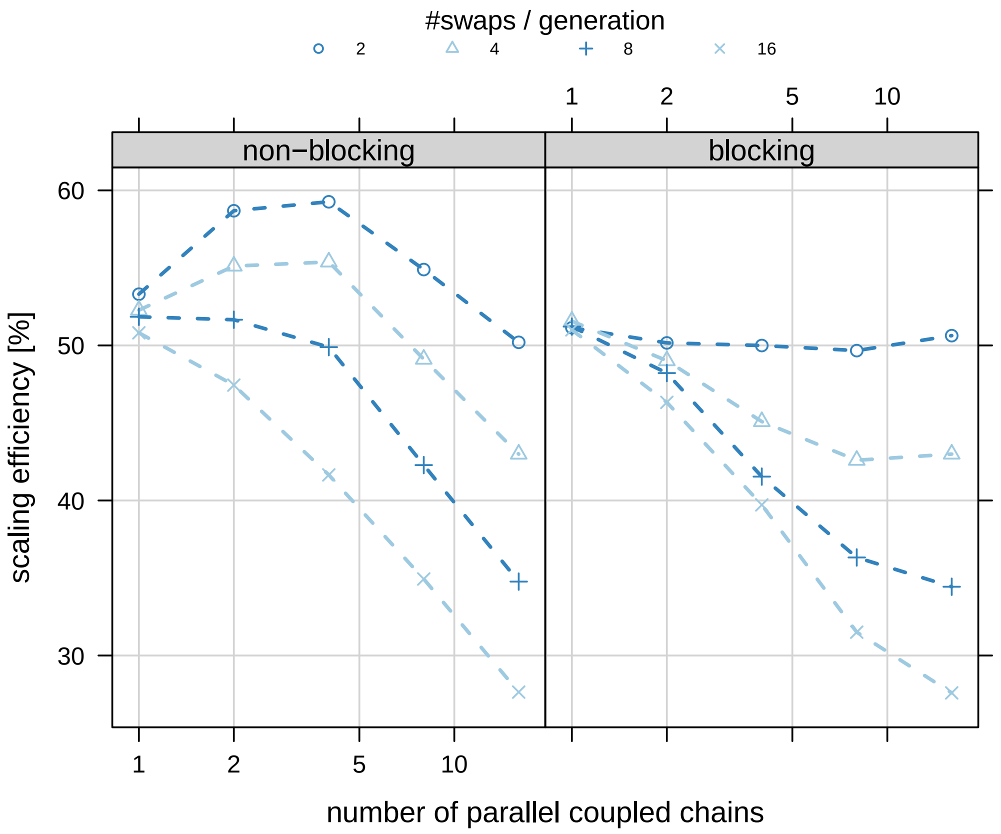

FIG. 5. Scaling efficiency of parallel execution of 16 Metropolis-coupled chains using 32 processes. Scaling efficiency for non-blocking algorithm *left* and blocking algorithm *right* for an increasing number of swaps per generation and an increasing number of chains executed in parallel (x-axis).

We executed a single chain using the different likelihood implementations in `ExaBayes` and `MrBayes` on various empirical single-partition small- to medium-size datasets (see **Tab.** 1) that were used for benchmarking proposal efficiency (Lakner *et al.*, 2008) and for evaluating the so-called bootstopping criterion (Pattengale *et al.*, 2009). In **Fig.** 6 we compare runtimes (averaged over 3 independent runs) relative to the non-SEV SSE version of `ExaBayes` (referred to as *exa-sse-nosev*). Compared to *exa-sse-nosev*, the SEV versions in `ExaBayes` perform similarly to the reference case, however are substantially faster for datasets with many taxa and a high proportion of missing data. Specifically, on the 994-taxon and 1,481-taxon datasets, the SEV implementations noticeably outperform any alternatives. For the latter case, the AVX version outperforms the reference case by more than a factor of 2.

As expected, the AVX version of the likelihood function is consistently faster than the SSE version (between 19.6% and 43.2%). With the exception of two amino acid datasets, the double-precision implementation of `BEAGLE` is consistently and noticeably slower than its `ExaBayes` counterpart. For three DNA datasets (with 994, 404 and 1,908 taxa), *exa-sse-nosev* is more than two times faster. The single-precision version

of BEAGLE in most cases is negligibly faster than the reference case, but never outperforms both AVX implementations in ExaBayes. On every dataset, the native likelihood implementation in MrBayes is faster than the BEAGLE alternatives. While the native likelihood implementation of MrBayes is consistently faster than *exa-sse-nosev*, it generally is slower than either the AVX version of either the SEV or the non-SEV likelihood implementation in ExaBayes, except for 5 datasets. Three of these are amino acid datasets, where the native MrBayes likelihood outperforms any alternative noticeably (more than two-fold in 2 cases).

Verification

We compare the results obtained by ExaBayes to those obtained by MrBayes (version 3.2.1) under the same settings (that is, same prior probability distributions and alignment data).

We simulated 240 DNA alignments using INDELible (Fletcher and Yang, 2009). Each alignment has 30 species and 2,000 characters which evolve according to a random GTR matrix along a randomly drawn phylogenetic tree. The tree is drawn according to a birth-death process with a birth-rate of 2.4, a death-rate of 1.1, a sampling-fraction of 0.2566 and a mutation-rate of 0.34. The five free rates for the GTR matrix (the sixth is scaled to 1.0) are uniformly drawn from $U(0.1,1.9)$. The state frequencies are uniformly drawn from $U(0.0,1.0)$ and normalized to sum up to 1.0.

We analyzed each alignment with ExaBayes and MrBayes under the same prior configuration. The priors for these analyses were chosen to be the default values implemented in MrBayes. By default, a uniform prior is used for the topology and the shape parameter (here: $U(0,200)$), a Dirichlet prior for the state frequencies ($D(1,1,1,1)$) and (GTR) substitution rates ($D(1,1,1,1,1,1)$). Branch length parameters have an exponential distribution ($Exp(10)$) as prior. We executed 8 independent runs with the sequential versions of ExaBayes and MrBayes employing no Metropolis-coupling and ran chains for 500,000 generations and sampling every 500-th generation.

In order to compare the topologies obtained by ExaBayes and MrBayes, we calculated the ASDSF score (Lakner *et al.*, 2008). On average, the ASDSF score for all 16 runs (8 from ExaBayes and 8 from MrBayes) of all 240 alignments was less than 0.00435. This result implies, that ExaBayes and MrBayes both converge towards the same posterior distribution for the tree topology (or more specifically to identical split frequencies). For continuous parameters, we compared the ratio of means and the ratio of standard deviations to determine whether both programs yield an identical marginal distribution (see **Tab.** 2). For all parameters, the ratio of means differed from 1 by less than 0.56% and the ratio of standard

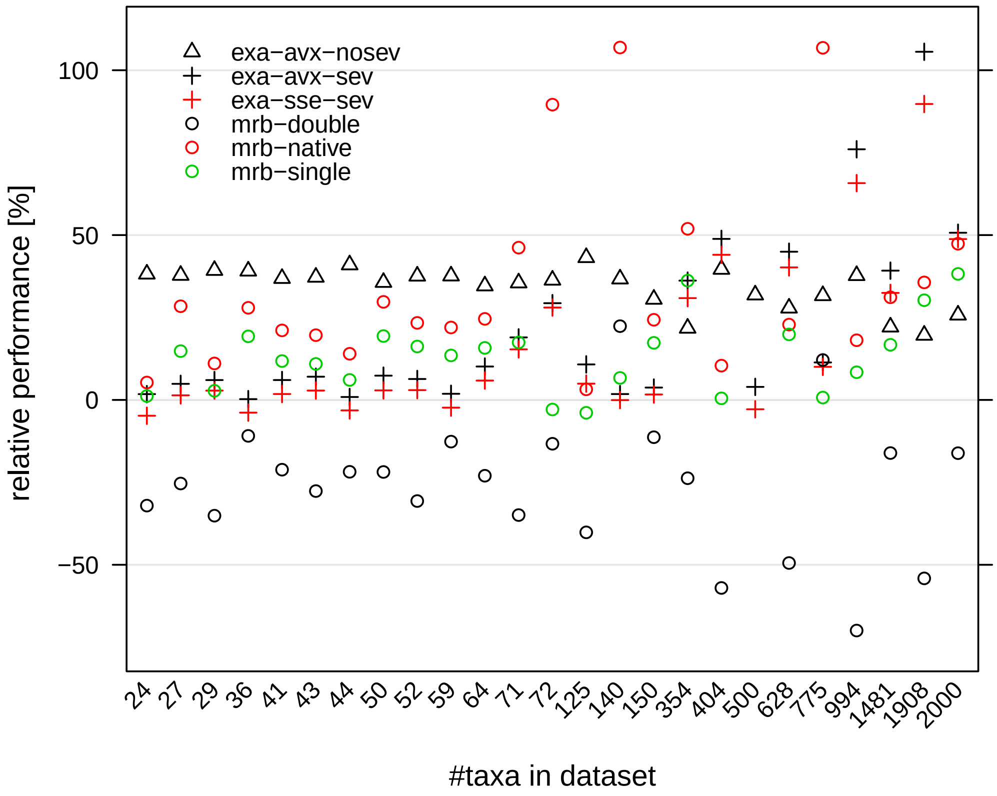

FIG. 6. Sequential runtime performance comparison between `ExaBayes` and `MrBayes` employing various implementations of the phylogenetic likelihood function. Runtimes are relative to the runtime of `ExaBayes` using the non-SEV (*nosev*) SSE implementation. Runtimes for `MrBayes` using either its native (*mrb-native*) implementation or a single- (*mrb-single*) or double-precision (*mrb-double*) implementation provided by `BEAGLE`.

deviations differed by less than 3.17%. This indicates that for continuous parameters, `MrBayes` and `ExaBayes` yield identical estimates.

taxa	characters	proportion of gaps [%]	unique site patterns	data type
24	14,190	0.26%	4,600	DNA
27	1,949	20.42%	934	DNA
29	2,520	30.57%	1246	DNA
36	1,812	0.02%	1,020	DNA
41	1,137	10.79%	768	DNA
43	1,660	11.03%	954	DNA
44	5,582	8.60%	2,788	DNA
49	1,149	3.29%	628	DNA
50	1,133	9.33%	489	DNA
52	2,157	25.19%	867	DNA
59	1,824	0.03%	1,037	DNA
64	1,008	21.21%	406	DNA
71	1,082	36.28%	445	DNA
72	32,883	52.09%	30,274	AA
125	29,149	32.72%	19,436	DNA
140	1,104	0.60%	1,041	AA
150	1,269	4.77%	1,130	DNA
354	460	14.71%	348	DNA
404	1,3158	78.92%	7,429	DNA
628	1,228	36.44%	1,033	DNA
775	4,519	19.35%	3,838	AA
994	5,533	71.39%	3,363	DNA
1,481	1,241	26.58%	1,241	DNA
1,908	1,424	58.38%	1,209	DNA
2,000	1,251	12.98%	1,251	DNA

Table 1. Real-world datasets for runtime comparisons listing number of taxa, number of characters, proportion of gaps in percent, number of unique site patterns and data type.

Parameter	Mean ASDSF:	ExaBayes	MrBayes	Combined	Max ASDSF:	ExaBayes	MrBayes	Combined
Topology		0.004194	0.004193	0.004348		0.008996	0.010181	0.009372
	Mean PSRF:	ExaBayes	MrBayes	Combined	Max PSRF:	ExaBayes	MrBayes	Combined
Log Likelihood		1.001121	1.001499	1.001313		1.008919	1.006595	1.006098
Tree length		1.000049	1.000489	1.000271		1.001470	1.004411	1.001960
alpha		1.000035	1.000127	1.000077		1.002147	1.002319	1.002047
$\bar{\pi}$		1.001669	1.004930	1.003336		-	-	-
$\pi(A)$		1.001331	1.004678	1.002947		1.007052	1.020463	1.010619
$\pi(C)$		1.001255	1.005177	1.003316		1.008266	1.020070	1.011627
$\pi(G)$		1.002303	1.004951	1.003720		1.060657	1.018953	1.043209
$\pi(T)$		1.001786	1.004913	1.003360		1.049445	1.019128	1.028040
$\bar{r}$		1.006873	1.005006	1.005898		-	-	-
$r(A,C)$		1.004198	1.005078	1.004919		1.205607	1.040531	1.114231
$r(A,G)$		1.004035	1.004719	1.004316		1.131113	1.032540	1.064979
$r(A,T)$		1.004724	1.005219	1.005068		1.224719	1.085399	1.116632
$r(C,T)$		1.003754	1.004905	1.004546		1.084591	1.056486	1.053862
$r(C,G)$		1.004557	1.004864	1.004627		1.151122	1.048051	1.073415
$r(G,T)$		1.019969	1.005248	1.011912		1.637836	1.050760	1.194479
	ESS	Mean Ratio:	Mean	Deviation	Max Ratio:	Mean	Deviation	
Log Likelihood	2082.83		0.0004 %	1.3268 %		0.0017 %	4.7812 %	
Tree length	4746.49		0.0211 %	1.0275 %		0.0815 %	4.0807 %	
alpha	4631.25		0.4670 %	0.7180 %		2.3236 %	2.7315 %	
$\bar{\pi}$	1824.76		0.1031 %	2.0707 %		1.9633 %	7.6879 %	
$\pi(A)$	1884.55		0.0793 %	2.0836 %		1.9703 %	7.2128 %	
$\pi(C)$	1844.54		0.1071 %	1.9810 %		1.0547 %	8.5399 %	
$\pi(G)$	1775.60		0.1337 %	2.1982 %		3.9599 %	8.1770 %	
$\pi(T)$	1794.35		0.0924 %	2.0199 %		0.8684 %	6.8221 %	
$\bar{r}$	1534.67		0.2723 %	2.1840 %		6.0016 %	21.3346 %	
$r(A,C)$	1544.38		0.2521 %	2.2135 %		4.1210 %	24.3313 %	
$r(A,G)$	1753.02		0.1609 %	1.8299 %		1.7217 %	7.9003 %	
$r(A,T)$	1732.28		0.2166 %	1.8639 %		3.6609 %	23.2922 %	
$r(C,T)$	1814.68		0.2330 %	1.9539 %		3.4961 %	20.1300 %	
$r(C,G)$	1811.53		0.2071 %	2.0731 %		4.7638 %	8.4670 %	
$r(G,T)$	552.11		0.5639 %	3.1698 %		18.2460 %	43.8868 %	

Table 2. List of average and maximum values of convergence statistics (ESS, PSRF and ASDSF) for continuous and discrete parameters estimated for 240 data sets using 8 independent `ExaBayes` and 8 independent `MrBayes` runs per dataset. Convergence statistics are shown for `ExaBayes` runs, `MrBayes` runs and the combination of all 16 runs (per dataset). Ratio of mean and ratio of standard deviation detail how much larger or smaller the respective statistic is for `ExaBayes` over `MrBayes`. $\pi(X)$ denotes state frequencies, $r(X,Y)$ denotes substitution rates, and $\bar{\pi}$ and $\bar{r}$ their respective mean values. For the topology parameter the ASDSF score is indicated analogously. Note that an ASDSF score below 0.01 is usually considered a strong indicator of convergence.

Reduced Memory Footprint

Description. For the recursive evaluation of the likelihood of a tree, memory requirements are largely dominated by conditional likelihood arrays (several entries for each character) that represent the conditional likelihood of a subtree. For n species, $(n-2)$ arrays have to be kept in memory, such that after the first evaluation further evaluations — that only require an update in some of the arrays (e.g., of a newly proposed branch length) — can be rapidly computed by reusing existing sub-tree likelihood arrays (that have not been affected by the proposal). In Bayesian phylogenetics, it is common to employ an additional set of likelihood arrays as backup: thus, if a proposal is rejected, the likelihood arrays for the previous state can be restored. When we apply Metropolis-coupling with c chains, we need one set of arrays for each chain, if p coupled chains are executed in parallel an additional backup set of likelihood arrays is needed for each parallel coupled chain. Thus, the total memory consumption is defined by a total of $(n-2)\cdot(c+p)$ arrays.

`ExaBayes` implements two orthogonal memory saving strategies. Firstly, `ExaBayes` offers an implementation of the likelihood function using subtree equality vectors (SEV) to omit the evaluation of sites in subtrees that entirely consist of undetermined or gap characters (Izquierdo-Carrasco *et al.*, 2011). The SEV-technique potentially reduces the length of several likelihood arrays proportional to the amount of missing data. Secondly, we allow to trade memory for runtime by reducing the number of arrays that are backed up. In case of rejection, arrays for which no backup array exists have to be re-computed, thus increasing runtime. We offer three settings:

1. No backup of arrays that are computed from two external (tip) nodes in the tree. Saves between 2 (comb-like tree) and $(n-3)$ arrays (balanced binary tree).
2. Only backup arrays that are computed from two inner nodes in the tree. Saves between $\lceil \frac{n}{2} \rceil$ (balanced binary tree) and $(n-3)$ arrays (comb-like tree).
3. Recompute all arrays (saves $(n-3)$ arrays, regardless of tree topology).

Evaluation. **Fig.** 7 illustrates the combination of both techniques to achieve a reduction in memory requirements of more than $\approx 60\%$ for a dataset with 1,908 species and 1,424 DNA characters (proportion of missing data: 58.38%). For the illustration of a memory versus runtime trade-off (see **Fig.** 8), we used the sequential version of `ExaBayes` and ran one chain for 10,000 generations for datasets of **Tab.** 1. Using strategy (1), we can reduce the memory requirements by more than 10% at the expense of about 5% additional runtime. For strategies (2) and (3), measurements for datasets separate into two groups: for one group strategy

(2) decreases memory requirements to 70%-80% (resp., 60%-70% for strategy (3)) of the original requirements while additional runtime expenses barely exceed 30% for strategy (2) (resp., ≈45%). This separation is due to the fact that many of the datasets only consist of a single gene and few taxa (see **Tab.** 1), thus have small requirements to begin with and constant overhead (as illustrated in **Fig.** 7) makes up for a relevant share of the overall memory consumption. We conclude that, our recomputation strategies are a flexible way to save up to 50% memory at the expense of less than 1.5× slow-down in analysis speed.

Highly Partitioned Analyses

Improvements. Alignments can be subdivided into partitions where distinct evolutionary models are assigned to each partition. When p processes are employed for data-level parallelism, we usually use a *cyclic data distribution* to assign every p-th character of a partition to process i using an offset of i. Processes evaluate their portion of the data and communicate their partial result to peer processes to determine the tree likelihood. Aside from the compute-intensive parallelized evaluation of the likelihood, pre-processing steps are necessary to determine in which order the likelihood arrays need to be evaluated and need to exponentiate the GTR matrix using its eigenvector/eigenvalue decomposition. Since each partition requires these steps, the sequential overhead can outweigh the distributed likelihood evaluation. If we assign entire partitions to processes using a multi-processor scheduling algorithm (Zhang and Stamatakis, 2012), the sequential overhead is distributed equally among processes (see **Fig.** 9 for an illustration of data distribution).

If all partitions are unlinked for a parameter, Bayesian inference on highly partitioned datasets only requires the evaluation of one partition per proposal, yet when executed in parallel an expensive communication step is necessary after evaluation. In these cases, we summarize all proposals of a kind (e.g., substitution rate proposals) to a *proposal set.* If a proposal set is drawn for a generation, an update is proposed for each component (possibly several linked partitions). Since the likelihood gain for each parameter is completely independent, we can evaluate the entire alignment using only one communication step. Proposals within a set are still tuned separately. Because of the independence of partitions, proposal sets do not decrease mixing efficiency. No modification of the proposal density ratio is necessary, since this scheme essentially represents a mixture of strictly ordered proposals (proposals within a set) and random proposals (whether a proposal set or another proposal such as a topological proposal is applied is determined randomly). Both strategies were discussed in the original introduction of the Metropolis-Hastings algorithm (Hastings, 1970) for MCMC.

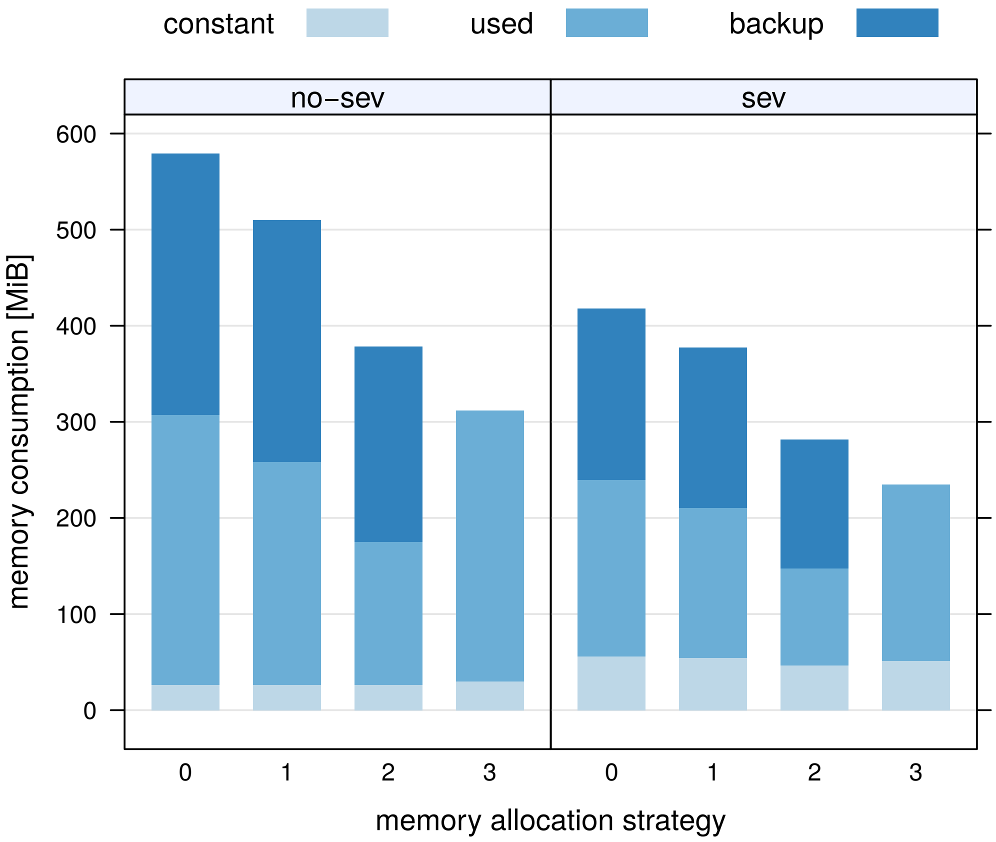

FIG. 7. Absolute memory consumption for 1 chain running on a dataset with 1,908 taxa and 1,424 DNA characters (gap proportion: 58.38%) using the SEV-technique (*sev*) or standard implementation of the likelihood function (*no-sev*) and the memory saving techniques discussed in the text (where 0 indicates that memory saving is disabled). Each measurement also indicates the proportion of memory *used* for likelihood computations, employed for *backup* arrays or *constant* memory overhead.

Evaluation For the evaluation of the aforementioned modifications, we simulated a 200 taxon alignment with 1000 partitions of 1000 bp. For various run settings, we ran a single chain employing 4 computing nodes (4 AMD Opteron processors with 12 cores) on our cluster at HITS using a total of 192 cores per run.

When proposal sets are used, a generation that evaluates the entire alignment is very expensive compared to a generation in the original scheme, where only one partition is evaluated. Thus, we normalized runtime by the total number of proposals executed in one run. Notice, that while generations are not comparable among runs where proposal sets are applied or not, the proportion of a proposal relative to the total number of proposals evaluated remains the same whether it is executed in a set or individually.

In **Fig.** 10 we examine normalized runtimes for run settings where we either use cyclic or partition data distribution combined with enabled or disabled proposal sets relative to the case where none of our two techniques are enabled. The left hand side of **Fig.** 10 shows runs for the more common case where branch lengths are linked across all partitions. In the usual proposal

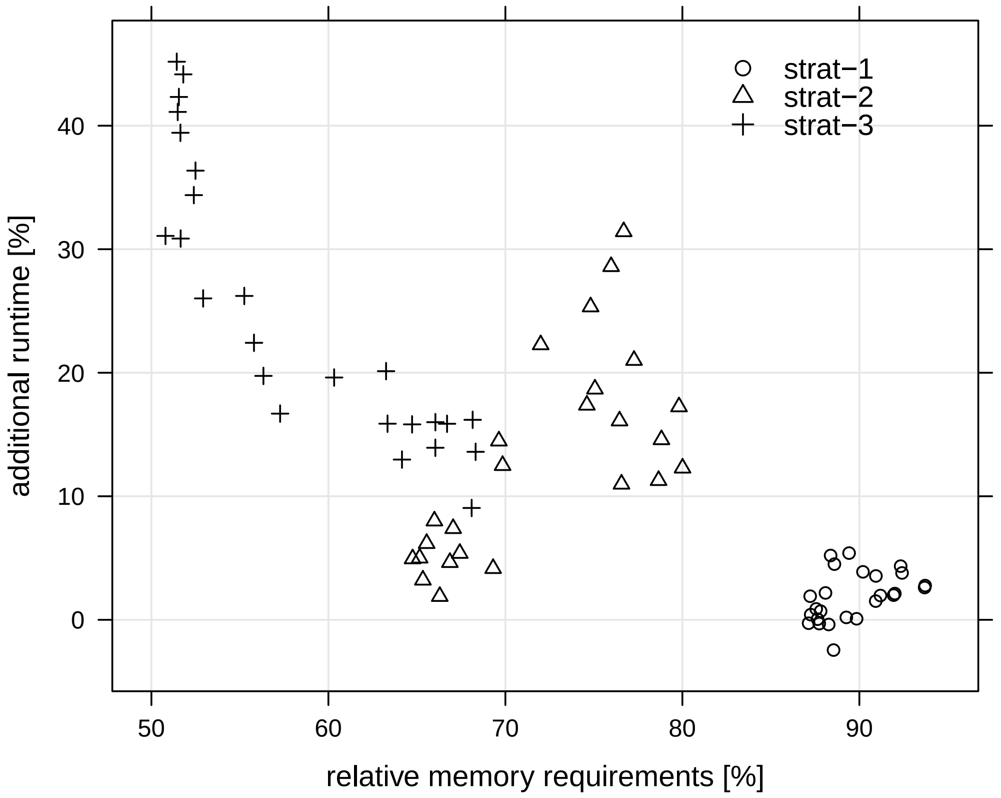

FIG. 8. Memory/runtime trade-off for different recomputation strategies (not using the SEV technique). Memory consumption (x-axis) and additional runtime (y-axis) relative to respective values for a runs without recomputation.

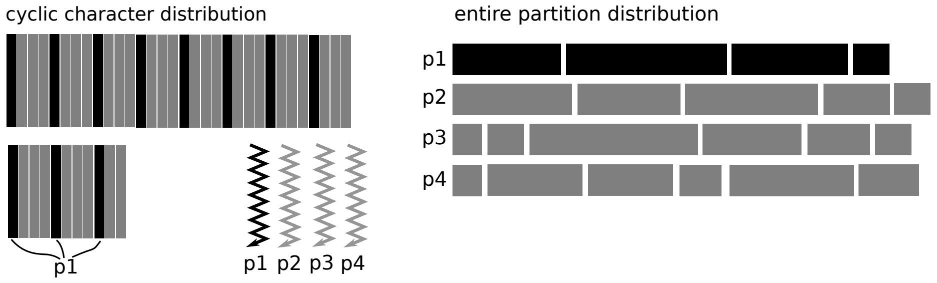

FIG. 9. Distribution schemes in `ExaBayes`: in the cyclic distribution scheme (*left*) with two partitions every 4th site is assigned to process p_1, whereas in the partition distribution scheme (*right*, illustrated with 21 partitions), entire partitions are assigned to processes such that the number of sites per process is similar.

mixture (employed in `MrBayes` and `ExaBayes`), proposals on topology and branch lengths are assigned a high weight, since they are usually the most difficult and in a different meaning of the word parameter rich (if we consider each possible inner edge a parameter). Thus, the proposal sets are only applied in 6.67% of all generations. This explains, why in **Fig.** 10, the partition distribution scheme improves runtime, although likelihood evaluation for single-partition proposals are executed sequentially. When both of our techniques are enabled, we achieve a speedup of 21.7 or in other words, we can compute more than 21× more proposals in the same time.

Combining the proposal set technique with partition data distribution has an even higher impact on normalized runtime, when branch lengths are unlinked across partitions (i.e., each gene has distinct branch lengths). Here, the combination of both of our techniques achieves a relative speedup of 87.0. In this case, proposal sets are used for all but the topological parameters (which account for 44.4% of generations). In contrast to the case with linked branch lengths, `ExaBayes` even becomes slower when partition distribution is enabled, but proposal sets are disabled. When repeating this experiment for more cpu-cores, we may expect that the performance gap further extends.

Whole-Genome Analysis

A whole genome DNA alignment was simulated using `INDELible` (Fletcher and Yang, 2009). The alignment has 200 species and 100,000,000 characters, separated into 100 equally long partitions (reminiscent of a chromosome partitioning scheme). Each partition was simulated to evolve according to a unique random GTR matrix along the same random phylogenetic tree. The five free rates for each of the 100 GTR matrices are uniformly drawn from $U(0.1,1.9)$ (the sixth is scaled to 1.0). The state frequencies are uniformly drawn from $U(0.0,1.0)$ and normalized to sum up to 1.0. The single tree is drawn according to a birth-death process with parameters: birth-rate$=2.4$ death-rate$=1.1$, sampling-fraction$=0.2566$ and mutation-rate$=0.34$. For the alignment, 1,000 individual small alignments with 100,000 characters each, were simulated using `INDELible`. Of these small alignments, 10 always share the same underlying GTR matrix and state frequencies. The 1,000 individual alignments were then concatenated (available under `http://exelixis-lab.org/material/simulated-genome.tbz`). The tree used for simulation is depicted in **Fig.** 11. Branch length in this tree range from $4.74482 \cdot 10^{-4}$ to $3.16679 \cdot 10^{-1}$.

For the analysis, 4 runs started from parsimony starting trees, 2 further runs from random starting trees. Branch lengths were linked across all partitions for all runs, and had exponentially distributed priors ($\lambda = 10$). Model parameters were unlinked across the partitions. State frequencies and GTR substitution rates had Dirichlet distributed priors ($D(1,1,1,1)$ and $D(1,1,1,1,1,1)$ respectively). Topology and shape parameter priors were uniform distributions (the latter $U(0,200)$).

The concatenated alignment file requires 21 GB disk space and the RAM requirements for likelihood arrays exceed 4.61 TB. We executed analyses at the LRZ using the SuperMUC computer. Thus, at the SuperMUC a minimum of 193 computing nodes (each 16 cpu-cores and 24 GB of accessible main memory) is needed to conduct a run. We used 4,096 CPU cores for runs starting from parsimony starting trees

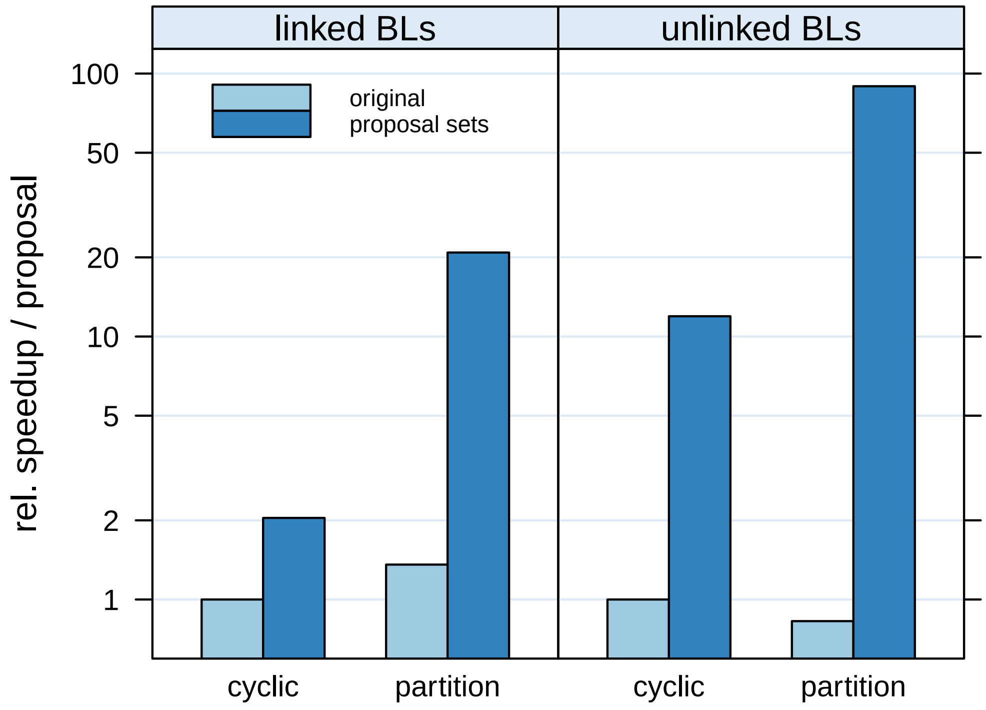

FIG. 10. Performance on Highly Partitioned Datasets: Speedup of per-proposal runtime for configurations relative to per-proposal runtime where neither partition data distribution nor proposal sets are employed. *Left:* branch lengths are linked across partitions, *right:* branch lengths are unlinked across partitions.

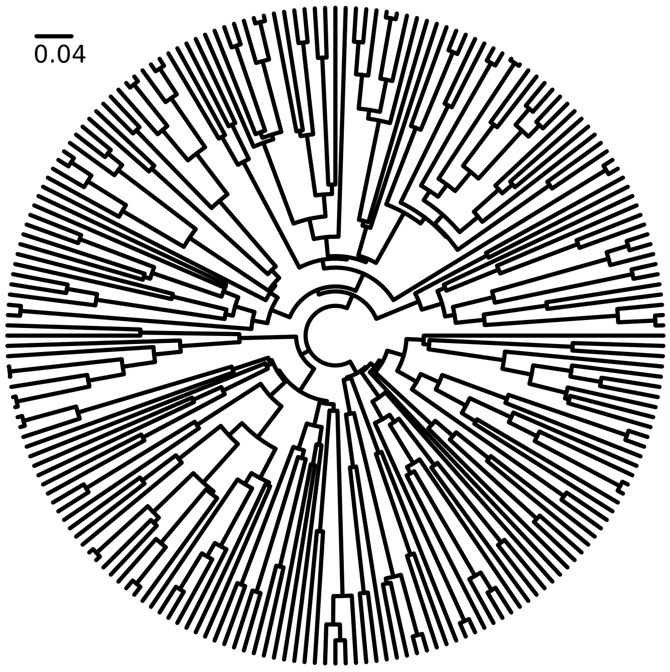

FIG. 11. The simulated tree comprising 200 species.

and 8,192 cores for runs using randomized starting trees. Given budget limitations of computational resources, we chose to run chains for 100,000 generations and extracted a sample from chains every 500 generations. Subsets of this alignment with 10,000 and 1,000,000 characters were run under the same conditions. Despite the low number of generations, clade posterior probabilities for chains that were started in a parsimony tree quickly converged against the simulated topology (see **Fig.** 12). The ASDSF of the two chains started in a random tree is critically high at the end of the simulation, because of the substantially extended burn-in phase. Still after ≈60,000 generations, the two chains started in random trees reached the correct tree. This indicates that convergence of the 4 other chains was not biased by using a parsimony starting tree. Furthermore, we conclude that using a parsimony tree instead of a random tree as initial state increases the burn-in length by a factor of 3×.

Fig. 13 showcases distributions of branch length samples extracted for 5 distinct splits across the three datasets. As expected, with increasing amount of data, the branch length samples become more accurate, respectively converge against the branch length used for simulating the alignment. A weak tendency to overestimate branch lengths is noticeable. **Fig.** 14 provides an overview of the degree of variation relative to the sample mean (expressed as the coefficient of variation that is defined as the ratio of standard deviation and mean of a distribution). For each split in the three datasets, we extracted the respective statistics after burnin and for splits with a posterior probability of at least 25%. The observation that more data leads to more accurate estimates also manifests in **Fig.** 14. With 10,000 characters, there is a strong correlation between mean and coefficient of variation of branch length samples. This relationship exists, because with shortest branch lengths, less evolutionary time passed and thus the probability of observing substitutions within this time frame is smaller. With enough data, coefficients of variation generally become smaller, however even with this amount of data, samples of the shortest branch lengths exhibit comparably large confidence intervals. We assume that coefficients of correlation already are at minimum for the dataset with 1,000,000 characters, since improvements with 100,000,000 characters are minor.

Because of the clear signal provided by the substantial amount of data, no Metropolis-coupling was necessary. While `ExaBayes` does not constrain the size of the analysis to be conducted, we discuss the SuperMUC as an example of the limits imposed by modern super-computers on analysis size. Given our simulated alignment, maximum recomputation and the 9,216 computing nodes available at the SuperMUC (where usually only 2,048 are available for a single job), Metropolis-coupling with 95

chains is theoretically feasible, if all coupled chains are executed in parallel. A dataset, that is merely one order of magnitude larger (i.e, 2,000 taxa or 1,000,000,000 characters), would allow for only 9 coupled chains if conducted on the entire SuperMUC.

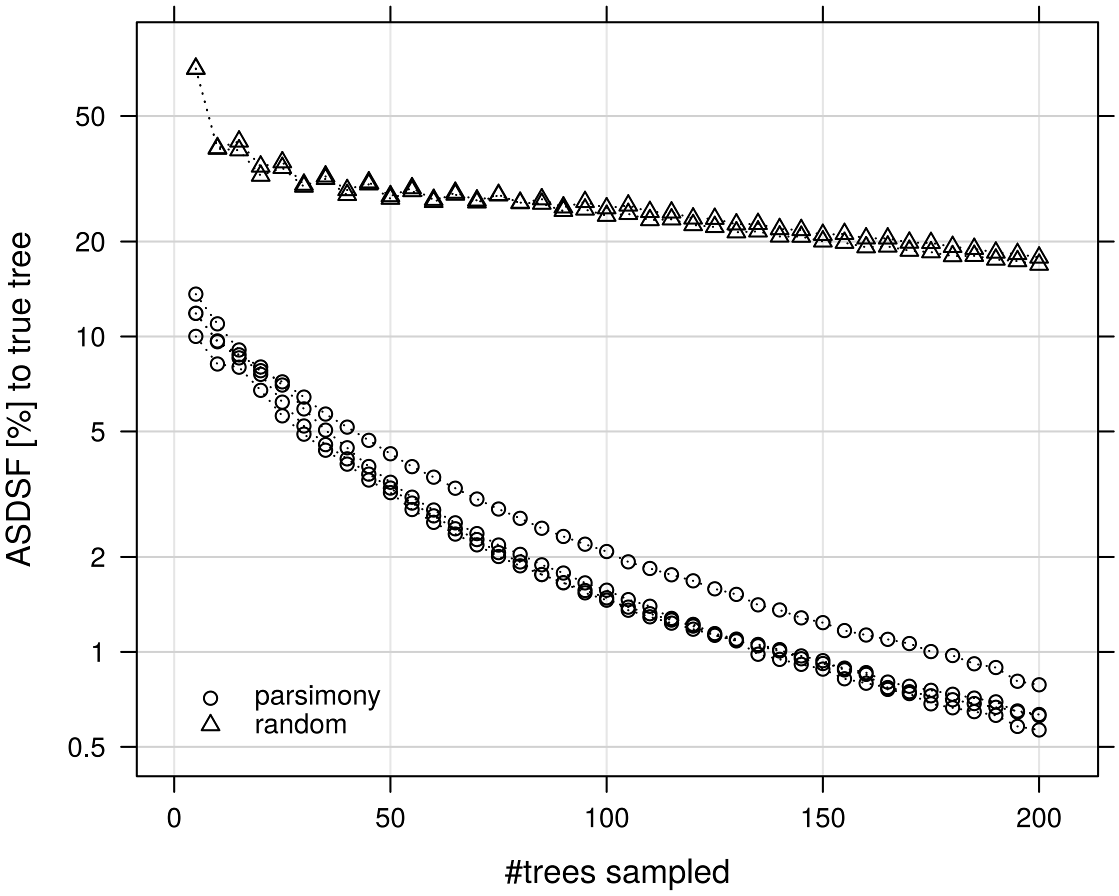

FIG. 12. Topological convergence for whole-genome analyses. ASDSF to true tree of 4 chains starting in parsimony trees and 2 chains starting with a randomized tree.

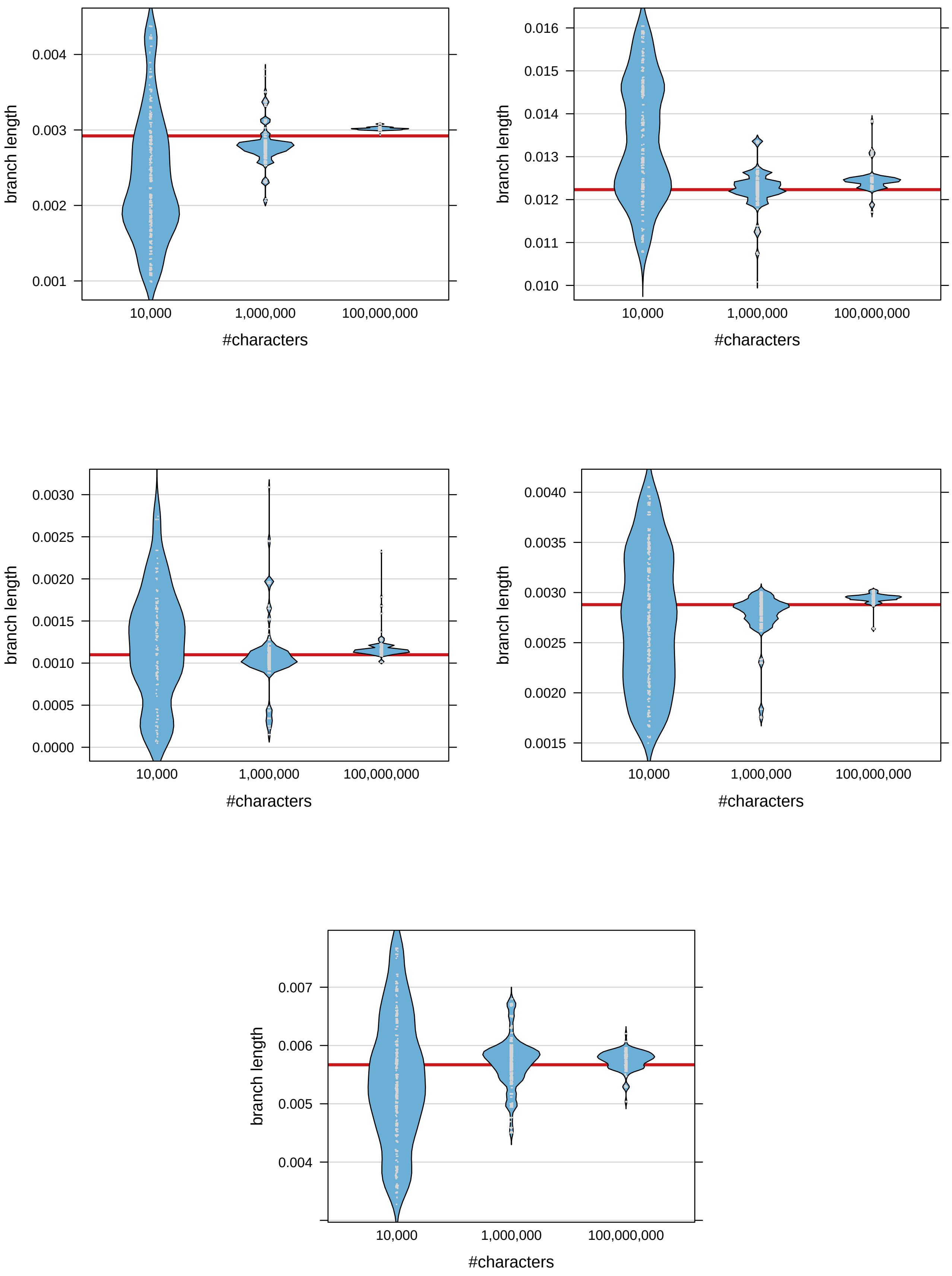

FIG. 13. Distributions of branch length samples (y-*axis*) from chains run on the whole-genome alignment and two truncated versions. Each figure corresponds to a distinct split in the tree used for simulation. Alignments size is increased in steps of 2 orders of magnitude (x-*axis*). Branch lengths in the tree used for simulation of the alignment in red.

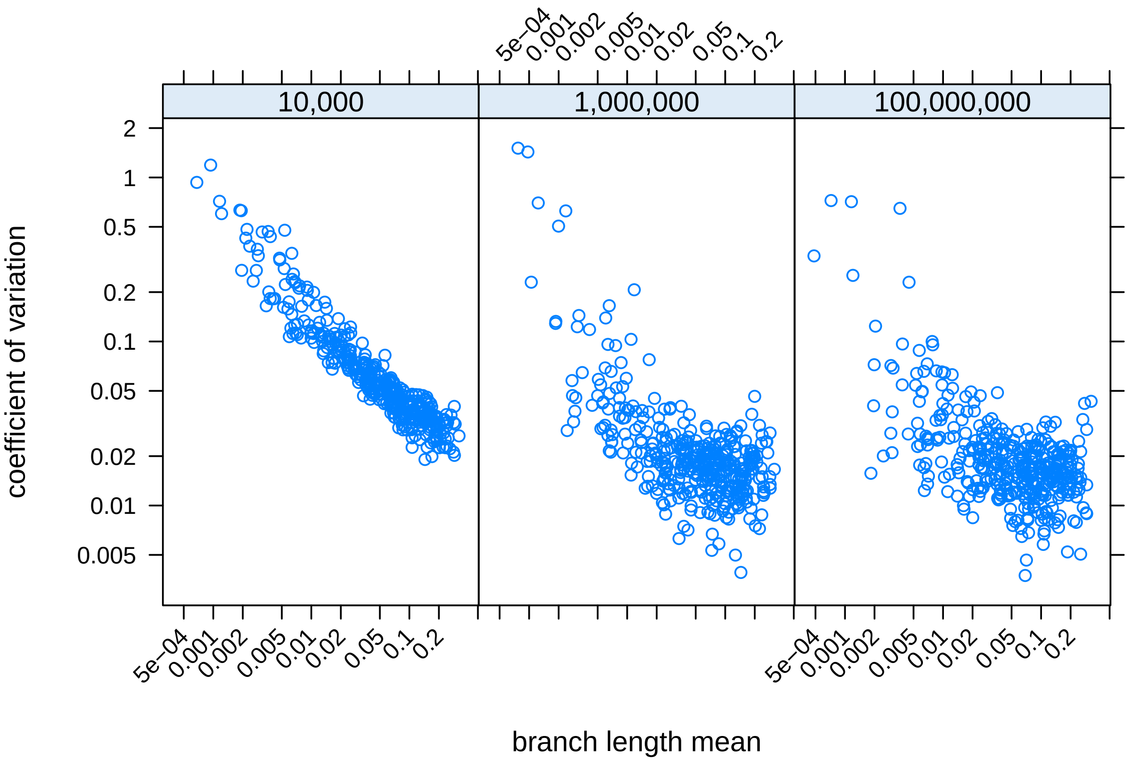

FIG. 14. Mean and coefficient of variation of branch length samples for the simulated whole genome dataset and its two truncated variants.

References

Alachiotis, N. and Stamatakis, A. 2011. Fpga acceleration of the phylogenetic parsimony kernel? In *Field Programmable Logic and Applications (FPL), 2011 International Conference on*, pages 417–422. IEEE.

Altekar, G., Dwarkadas, S., Huelsenbeck, J. P., and Ronquist, F. 2004. Parallel Metropolis coupled Markov chain Monte Carlo for Bayesian phylogenetic inference. *Bioinformatics (Oxford, England)*, 20(3): 407–15.

Atchadé, Y. F., Roberts, G. O., and Rosenthal, J. S. 2010. Towards optimal scaling of metropolis-coupled Markov chain Monte Carlo. *Statistics and Computing*, 21(4): 555–568.

Ayres, D. L., Darling, A., Zwickl, D. J., Beerli, P., Holder, M. T., Lewis, P. O., Huelsenbeck, J. P., Ronquist, F., Swofford, D. L., Cummings, M. P., Rambaut, A., and Suchard, M. a. 2012. BEAGLE: an application programming interface and high-performance computing library for statistical phylogenetics. *Systematic biology*, 61(1): 170–3.

Berger, S. A. and Stamatakis, A. 2010. Accuracy and performance of single versus double precision arithmetics for maximum likelihood phylogeny reconstruction. In *Parallel Processing and Applied Mathematics*, pages 270–279. Springer.

DellAmpio, E., Meusemann, K., Szucsich, N. U., Peters, R. S., Meyer, B., Borner, J., Petersen, M., Aberer, A. J., Stamatakis, A., Walzl, M. G., *et al.* 2014. Decisive data sets in phylogenomics: Lessons from studies on the phylogenetic relationships of primarily wingless insects. *Molecular biology and evolution*, 31(1): 239–249.

Fitch, W. M., Margoliash, E., *et al.* 1967. Construction of phylogenetic trees. *Science*, 155(760): 279–284.

Fletcher, W. and Yang, Z. 2009. INDELible: a flexible simulator of biological sequence evolution. *Molecular biology and evolution*, 26(8): 1879–88.

Geyer, C. J. 1992. *Markov chain Monte Carlo maximum likelihood*. Defense Technical Information Center.

Hastings, W. K. 1970. Monte carlo sampling methods using markov chains and their applications. *Biometrika*, 57(1): 97–109.

Izquierdo-Carrasco, F., Smith, S. a., and Stamatakis, A. 2011. Algorithms, data structures, and numerics for likelihood-based phylogenetic inference of huge trees. *BMC bioinformatics*, 12(1): 470.

Lakner, C., van der Mark, P., Huelsenbeck, J. P., Larget, B., and Ronquist, F. 2008. Efficiency of Markov chain Monte Carlo tree proposals in Bayesian phylogenetics. *Systematic biology*, 57(1): 86–103.

Le, S. Q. and Gascuel, O. 2008. An improved general amino acid replacement matrix. *Molecular biology and evolution*, 25(7): 1307–1320.

Lewis, P. O. 2003. Ncl: a c++ class library for interpreting data files in nexus format. *Bioinformatics*, 19(17): 2330–2331.

Pattengale, N. D., Alipour, M., Bininda-Emonds, O. R., Moret, B. M., and Stamatakis, A. 2009. How many bootstrap replicates are necessary? In *Research in Computational Molecular Biology*, pages 184–200. Springer.

Salmon, J. K., Moraes, M. A., Dror, R. O., and Shaw, D. E. 2011. Parallel random numbers: as easy as 1, 2, 3. In *High Performance Computing, Networking, Storage and Analysis (SC), 2011 International Conference for*, pages 1–12. IEEE.

Stamatakis, A. 2014. Raxml version 8: A tool for phylogenetic analysis and post-analysis of large phylogenies. *Bioinformatics*.

Stamatakis, A. and Aberer, A. J. 2013. Novel parallelization schemes for large-scale likelihood-based phylogenetic inference. In *IPDPS*, pages 1195–1204.

Tavaré, S. 1986. Some probabilistic and statistical problems in the analysis of dna sequences. *Lect. Math. Life Sci*, 17: 57–86.

Whelan, S. and Goldman, N. 2001. A general empirical model of protein evolution derived from multiple protein families using a maximum-likelihood approach. *Molecular biology and evolution*, 18(5): 691–699.

Yang, Z. 1994. Maximum likelihood phylogenetic estimation from dna sequences with variable rates over sites: approximate methods. *Journal of Molecular evolution*, 39(3): 306–314.

Zhang, J. and Stamatakis, A. 2012. The multi-processor scheduling problem in phylogenetics. In *Parallel and Distributed Processing Symposium Workshops & PhD Forum (IPDPSW), 2012 IEEE 26th International*, pages 691–698. IEEE.
